# Supplementary material for: Health services costs for lung cancer care in Australia: Estimates from the 45 and Up Study
Source: PLoS One. 2020 Aug 31;15(8):e0238018. doi: 10.1371/journal.pone.0238018 (PMC7458299; doi:10.1371/journal.pone.0238018)
Supplement: S1 File — Table A. Summary statistics for excess costs overall, by phase of care and by month for incident lung cancer cases diagnosed 2006–2013. * For cases alive at the start of each time period. Costs reported in Australian dollars for the year 2013. Table B. Codes for identifying lung cancer-related treatment with surgery, chemotherapy or radiotherapy*. * Records included were from one month prior to cancer registry diagnosis date onwards. APDC: Admitted Patient Data Collection; ATC: Anatomical Therapeutic Chemical Classification System; MBS: Medicare Benefits Schedule; PBS: Pharmaceutical Benefits Scheme. Table C. Multivariable analysis of costs for lung cancer cases diagnosed 2006–2013. * Excludes cases with missing values for any of the listed variables and any cost outliers. Results for each variable are adjusted for all listed variables. Table D. Summary mean excess costs for eligible incident lung cancer cases diagnosed 2006–2013, with proportion of the excess costs by source. * Hospital-based services combines Admitted Patient Data Collection and Emergency Department costs, the latter accounted for 1–3% of costs for each time period except in the 3 months before diagnosis (5–6%). Costs reported in 2013 Australian dollars. MBS: Medicare Benefits Schedule; PBS: Pharmaceutical Benefits Scheme. Table E. Summary excess costs by phase of care and selected tumour characteristics, for eligible incident lung cancer cases diagnosed 2006–2013. NSCLC: non-small cell lung cancer, includes “Other specified carcinoma”; SCLC: small cell lung cancer. * Spread of disease at diagnosis as reported by the New South Wales Cancer Registry. Some of these numbers are relatively small and estimates may be somewhat unstable. Table F. Summary excess costs by in the year prior to diagnosis, by selected tumour characteristics, for eligible incident lung cancer cases diagnosed 2006–2013. NSCLC: non-small cell lung cancer, includes “Other specified carcinoma”; SCLC: small cell lung cancer. * Spre [file pone.0238018.s001.docx]

**Health services costs for lung cancer care in Australia: Estimates from the 45 and Up Study**

**Supporting information**

**Table A. Summary statistics for excess costs overall, by phase of care and by month for incident lung cancer cases diagnosed 2006-2013.**

|  | **No. of cases**  **included** | **Mean** | **Standard deviation** | **Median** | **Quartile 1**  **(25^th^ centile)** | **Quartile 3**  **(75^th^ centile)** |
| --- | --- | --- | --- | --- | --- | --- |
| “Average case” costs 1 year before to 3 years after diagnosis | 916 | 51,944 | 50,264 | 40,336 | 20,319 | 70,994 |
| Phase of care |  |  |  |  |  |  |
| Initial | 485 | 27,052 | 24,905 | 21,850 | 8,804 | 37,683 |
| Continuing (per year) | 313 | 7,797 | 20,786 | 3,606 | -1,310 | 12,896 |
| Terminal | 788 | 41,398 | 36,758 | 31,880 | 16,524 | 58,956 |
| By month relative to diagnosis* |  |  |  |  |  |  |
| Month 12 before diagnosis | 994 | -10 | 2,741 | -30 | -251 | 171 |
| Month 11 before diagnosis | 994 | -62 | 2,060 | -23 | -237 | 162 |
| Month 10 before diagnosis | 994 | 155 | 2,622 | -33 | -212 | 179 |
| Month 9 before diagnosis | 994 | 132 | 2,312 | -28 | -195 | 172 |
| Month 8 before diagnosis | 994 | -66 | 1,964 | -15 | -220 | 177 |
| Month 7 before diagnosis | 994 | 31 | 2,721 | -31 | -231 | 154 |
| Month 6 before diagnosis | 994 | 23 | 2,377 | -12 | -219 | 212 |
| Month 5 before diagnosis | 994 | 3 | 2,635 | -34 | -230 | 202 |
| Month 4 before diagnosis | 994 | 143 | 3,213 | -16 | -208 | 206 |
| Month 3 before diagnosis | 994 | 343 | 3,261 | 27 | -183 | 338 |
| Month 2 before diagnosis | 994 | 645 | 3,894 | 94 | -144 | 598 |
| Month 1 before diagnosis | 994 | 2,637 | 7,924 | 1,021 | 253 | 2,764 |
| Month 1 after diagnosis | 994 | 11,866 | 14,896 | 7,410 | 2,396 | 17,722 |
| Month 2 after diagnosis | 908 | 5,986 | 9,641 | 2,378 | 349 | 8,557 |
| Month 3 after diagnosis | 832 | 4,538 | 7,568 | 1,752 | 231 | 6,348 |
| Month 4 after diagnosis | 761 | 3,515 | 7,492 | 1,032 | 91 | 4,127 |
| Month 5 after diagnosis | 710 | 2,883 | 6,118 | 698 | 34 | 3,443 |
| Month 6 after diagnosis | 674 | 3,221 | 7,562 | 679 | 34 | 3,367 |
| Month 7 after diagnosis | 640 | 2,618 | 6,318 | 511 | -29 | 3,307 |
| Month 8 after diagnosis | 608 | 2,587 | 6,219 | 311 | -31 | 3,176 |
| Month 9 after diagnosis | 584 | 2,287 | 5,685 | 359 | -42 | 2,894 |
| Month 10 after diagnosis | 554 | 2,429 | 5,467 | 365 | -10 | 3,193 |
| Month 11 after diagnosis | 524 | 1,854 | 5,125 | 277 | -66 | 1,700 |
| Month 12 after diagnosis | 502 | 2,414 | 6,456 | 261 | -72 | 2,478 |
| Month 13 after diagnosis | 484 | 1,833 | 4,696 | 274 | -48 | 1,845 |
| Month 14 after diagnosis | 464 | 1,706 | 5,694 | 203 | -59 | 1,062 |
| Month 15 after diagnosis | 449 | 1,660 | 4,441 | 221 | -94 | 1,182 |
| Month 16 after diagnosis | 428 | 1,497 | 4,522 | 184 | -147 | 1,168 |
| Month 17 after diagnosis | 410 | 1,996 | 6,293 | 150 | -131 | 1,596 |
| Month 18 after diagnosis | 395 | 1,651 | 4,946 | 152 | -111 | 1,041 |
| Month 19 after diagnosis | 380 | 1,734 | 5,985 | 96 | -130 | 783 |
| Month 20 after diagnosis | 367 | 1,395 | 5,269 | 85 | -133 | 935 |
| Month 21 after diagnosis | 359 | 1,769 | 5,938 | 119 | -126 | 728 |
| Month 22 after diagnosis | 352 | 1,370 | 4,039 | 113 | -110 | 806 |
| Month 23 after diagnosis | 341 | 1,257 | 4,698 | 63 | -149 | 476 |
| Month 24 after diagnosis | 334 | 1,255 | 4,591 | 105 | -148 | 1,029 |

** For cases alive at the start of each time period.*

*Costs reported in Australian dollars for the year 2013.*

**Table B. Codes for identifying lung cancer-related treatment with surgery, chemotherapy or radiotherapy*.**

| **Treatment type** | **Data type** | **Codes** |
| --- | --- | --- |
| Surgery | APDC procedure | 38438-00,38438-01,38438-02,38440-00,38440-01,38441-00, 38441-01,90169-00 |
|  | MBS item | 38438,38440,38441 |
| Chemotherapy | APDC procedure | 13915-00,13918-00,13921-00,13924-00,13927-00,90760-00, 90767-00,90768-00,96199-00,96200-00,96201-00,96203-00, 96204-00 |
|  | APDC diagnosis | Z51.1, Z51.2 |
|  | MBS item | 13915,13918,13921,13924,13927,13930,13933,13936,13945 |
|  | PBS ATC code | All codes starting with “L01” (L01AA01-L01XX41) |
| Radiotherapy | APDC procedure | 15100-00,15203-00,15204-00,15207-00,15208-00,15224-00, 15239-00,15254-00,15269-00,15506-01,15506-02,15518-00, 15521-00,15524-00,15550-00,15600-00,15600-01,90765-00, 90765-01,90765-02,90765-03 |
|  | APDC diagnosis | Z51.0 |
|  | MBS item | 15000-15399, 15500-15600, 15700-15899 |

** Records included were from one month prior to cancer registry diagnosis date onwards.*

*APDC: Admitted Patient Data Collection; ATC: Anatomical Therapeutic Chemical Classification System; MBS: Medicare Benefits Schedule; PBS: Pharmaceutical Benefits Scheme.*

**Table C. Multivariable analysis of costs for lung cancer cases diagnosed 2006-2013.**

| **Category** | **“Average case” costs** | **Initial phase** | **Continuing phase** | **Terminal phase** |
| --- | --- | --- | --- | --- |
| No. of cases included | 915 | 483 | 310 | 783 |
| Sex | *p=0.812* | *p=0.947* | *p=0.056* | *p=0.777* |
| Female | 0.0047 | -0.001 | 0.0346 | -0.0044 |
| Male | (reference) | (reference) | (reference) | (reference) |
| Age at diagnosis (years) | *p<0.0001* | *p=0.001* | *p=0.005* | *p<0.0001* |
| 45-59 | 0.0382 | -0.006 | 0.0516 | 0.0849 |
| 60-69 | (reference) | (reference) | (reference) | (reference) |
| 70-79 | -0.0861 | -0.0373 | -0.0397 | -0.0442 |
| 80+ | -0.2390 | -0.0974 | -0.0380 | -0.1748 |
| Tumour histology | *p=0.067* | *p=0.540* | *p=0.400* | *p=0.322* |
| Small cell carcinoma | -0.0321 | -0.0111 | 0.0079 | 0.0015 |
| Non-small cell carcinoma | (reference) | (reference) | (reference) | (reference) |
| Other specified carcinoma | -0.0657 | -0.0311 | -0.0271 | 0.0032 |
| Other/Unspecified | -0.0693 | -0.0251 | -0.0737 | -0.0592 |
| Stage at diagnosis | *p=0.002* | *p<0.0001* | *p=0.001* | *p<0.0001* |
| Localised | -0.0230 | -0.0345 | -0.0999 | 0.0307 |
| Regional | 0.0027 | -0.0144 | -0.0984 | -0.0317 |
| Distant metastases | (reference) | (reference) | (reference) | (reference) |
| Unknown | -0.1066 | -0.1477 | -0.0782 | -0.1160 |
| Year of diagnosis | *p=0.576* | *p=0.755* | *p=0.172* | *p=0.973* |
| 2006-2008 | 0.0323 | -0.0029 | 0.0561 | -0.0026 |
| 2009 | 0.0217 | -0.0246 | 0.0223 | 0.0164 |
| 2010 | (reference) | (reference) | (reference) | (reference) |
| 2011 | -0.0007 | -0.0278 | 0.0443 | 0.0053 |
| 2012 | -0.0215 | -0.0316 | -0.0075 | 0.0105 |
| 2013 | -0.0114 | -0.0102 | -0.0038 | 0.0005 |
| Smoking status | *p<0.0001* | *p=0.168* | *p=0.005* | *p=0.003* |
| Never / Ex quit >15yrs | (reference) | (reference) | (reference) | (reference) |
| Ex-smoker quit <=15yrs | -0.0515 | -0.0165 | -0.0129 | -0.0257 |
| Current smoker | -0.1060 | -0.0376 | -0.0738 | -0.0657 |
| Remoteness of place of residence | *p=0.052* | *p=0.078* | *p=0.280* | *p=0.058* |
| Major cities | (reference) | (reference) | (reference) | (reference) |
| Inner regional | -0.0035 | -0.0304 | -0.0274 | 0.0060 |
| Outer regional/(Very) Remote | -0.0689 | -0.0447 | 0.0074 | -0.0479 |
| Socioeconomic quintile | *p=0.013* | *p=0.792* | *p=0.520* | *p=0.462* |
| Most disadvantaged quintile | 0.0740 | 0.0116 | 0.0273 | 0.0364 |
| Quintile 2 | 0.0593 | -0.0069 | 0.0088 | 0.0261 |
| Quintile 3 | 0.1225 | 0.0220 | 0.0293 | 0.0529 |
| Quintile 4 | 0.0349 | 0.0071 | -0.0181 | 0.0215 |
| Least disadvantaged quintile | (reference) | (reference) | (reference) | (reference) |
| Health insurance status | *p=0.277* | *p=0.059* | *p=0.615* | *p=0.013* |
| Private insurance | (reference) | (reference) | (reference) | (reference) |
| Concession card | -0.0224 | -0.0414 | 0.0065 | -0.0483 |
| None | -0.0422 | -0.0311 | -0.0184 | -0.0475 |

** Excludes cases with missing values for any of the listed variables and any cost outliers.*

*Results for each variable are adjusted for all listed variables.*

**Table D. Summary mean excess costs for eligible incident lung cancer cases diagnosed 2006-2013, with proportion of the excess costs by source.**

| **Cost category** | **No. of cases included** | **Mean excess cost per case ($)** | **Hospital-based care* (%)** | **MBS (%)** | **PBS (%)** |
| --- | --- | --- | --- | --- | --- |
| Monthly costs around diagnosis |  |  |  |  |  |
| Month 4 before diagnosis | 994 | 143 | 25% | 55% | 20% |
| Month 3 before diagnosis | 994 | 343 | 59% | 32% | 8% |
| Month 2 before diagnosis | 994 | 645 | 56% | 39% | 5% |
| Month 1 before diagnosis | 994 | 2,637 | 66% | 32% | 2% |
| Month 1 after diagnosis | 994 | 11,866 | 83% | 15% | 2% |
| Month 2 after diagnosis | 908 | 5,986 | 77% | 16% | 7% |
| Month 3 after diagnosis | 832 | 4,538 | 68% | 16% | 16% |
| Month 4 after diagnosis | 761 | 3,515 | 65% | 16% | 20% |
| Month 5 after diagnosis | 710 | 2,883 | 61% | 17% | 22% |
| Month 6 after diagnosis | 674 | 3,221 | 63% | 15% | 21% |
| Month 7 after diagnosis | 640 | 2,618 | 57% | 17% | 26% |
| Month 8 after diagnosis | 608 | 2,587 | 55% | 12% | 33% |
| Month 9 after diagnosis | 584 | 2,287 | 58% | 13% | 29% |
| Month 10 after diagnosis | 554 | 2,429 | 64% | 13% | 24% |
| Month 11 after diagnosis | 524 | 1,854 | 55% | 18% | 28% |
| Month 12 after diagnosis | 502 | 2,414 | 63% | 13% | 24% |
| Month 13 after diagnosis | 484 | 1,833 | 51% | 17% | 32% |
| Month 14 after diagnosis | 464 | 1,706 | 54% | 16% | 31% |
| Month 15 after diagnosis | 449 | 1,660 | 55% | 14% | 31% |
| Month 16 after diagnosis | 428 | 1,497 | 57% | 16% | 27% |
| Month 17 after diagnosis | 410 | 1,996 | 58% | 15% | 27% |
| Month 18 after diagnosis | 395 | 1,651 | 57% | 17% | 26% |
| Monthly costs at the end of life |  |  |  |  |  |
| Month 6 before death | 476 | 3,585 | 58% | 16% | 26% |
| Month 5 before death | 508 | 3,930 | 62% | 15% | 23% |
| Month 4 before death | 559 | 4,364 | 69% | 14% | 17% |
| Month 3 before death | 628 | 4,909 | 73% | 13% | 14% |
| Month 2 before death | 703 | 6,577 | 81% | 10% | 10% |
| Final month of life | 787 | 15,089 | 92% | 5% | 3% |
| Phase of care |  |  |  |  |  |
| Initial (up to 1 year after diagnosis) | 485 | 27,052 | 61% | 22% | 17% |
| Continuing care (per year) | 313 | 7,797 | 22% | 24% | 54% |
| Terminal (up to 1 year) | 788 | 41,398 | 76% | 11% | 13% |
| “Average case” |  |  |  |  |  |
| 1 year before to 3 years after diagnosis | 916 | 51,944 | 67% | 16% | 16% |

** Hospital-based services combines Admitted Patient Data Collection and Emergency Department costs, the latter accounted for 1-3% of costs for each time period except in the 3 months before diagnosis (5-6%).*

*Costs reported in 2013 Australian dollars. MBS: Medicare Benefits Schedule; PBS: Pharmaceutical Benefits Scheme.*

**Table E. Summary excess costs by phase of care and selected tumour characteristics, for eligible incident lung cancer cases diagnosed 2006-2013.**

|  | **Initial treatment phase** | | | **Continuing care phase** | | | **Terminal care phase** | | |
| --- | --- | --- | --- | --- | --- | --- | --- | --- | --- |
|  | **No. of cases** | **Mean cost** | **Standard deviation** | **No. of cases** | **Mean cost** | **Standard deviation** | **No. of cases** | **Mean cost** | **Standard deviation** |
| SCLC | 35 | $26,472 | $24,163 | 16 | $7,734 | $12,293 | 90 | $43,599 | $33,959 |
| NSCLC by stage* |  |  |  |  |  |  |  |  |  |
| Localised | 156 | $27,679 | $21,590 | 131 | $4,705 | $15,630 | 72 | $53,679 | $46,751 |
| Regional | 129 | $30,989 | $25,507 | 88 | $5,995 | $24,781 | 141 | $42,718 | $43,229 |
| Distant metastases | 114 | $32,857 | $27,983 | 49 | $18,729 | $25,769 | 374 | $45,301 | $32,104 |
| Unknown | 38 | $10,859 | $17,243 | 22 | $9,272 | $19,414 | 67 | $25,324 | $32,273 |

*NSCLC: non-small cell lung cancer, includes “Other specified carcinoma”; SCLC: small cell lung cancer.*

** Spread of disease at diagnosis as reported by the New South Wales Cancer Registry.*

*Note: Some of these numbers are relatively small and estimates may be somewhat unstable.*

**Table F. Summary excess costs in the year prior to diagnosis, by selected tumour characteristics, for eligible incident lung cancer cases diagnosed 2006-2013.**

|  | **No. of cases** | **Mean cost** | **Standard deviation** |
| --- | --- | --- | --- |
| SCLC | 102 | $4,289 | $13,155 |
| NSCLC by stage* |  |  |  |
| Localised | 180 | $6,010 | $19,023 |
| Regional | 188 | $4,196 | $16,446 |
| Distant metastases | 397 | $2,427 | $15,216 |
| Unknown | 80 | $4,606 | $16,779 |

*NSCLC: non-small cell lung cancer, includes “Other specified carcinoma”; SCLC: small cell lung cancer.*

** Spread of disease at diagnosis as reported by the New South Wales Cancer Registry.*

**Table G. Number of cases in each phase and proportion having anti-cancer treatment, by selected tumour characteristics, for eligible incident lung cancer cases diagnosed 2006-2013.**

|  | **No. in initial phase** | **No. in continuing phase** | **No. in terminal phase** | **% had anti-cancer treatment (95% CI)^a^** |
| --- | --- | --- | --- | --- |
| Small cell lung cancer | 35 | 16 | 90 | 86.8% (80.2%-93.4%) |
| NSCLC by stage^b^ |  |  |  |  |
| Localised | 156 | 131 | 72 | 88.3% (83.4%-93.3%) |
| Regional | 129 | 88 | 141 | 88.8% (84.2%-93.4%) |
| Distant metastases | 114 | 49 | 374 | 71.3% (66.7%-75.9%) |
| Unknown | 38 | 22 | 67 | 56.2% (47.6%-64.9%) |
| NSCLC by age at diagnosis | |  |  |  |
| 45-59 | 60 | 45 | 77 | 89.3% (83.4%-95.2%) |
| 60-69 | 150 | 105 | 192 | 83.9% (79.3%-88.4%) |
| 70-79 | 162 | 107 | 226 | 78.7% (74.0%-83.3%) |
| 80+ | 65 | 33 | 159 | 53.9% (46.8%-61.0%) |
| NSCLC by sex |  |  |  |  |
| Female | 220 | 155 | 274 | 77.4% (73.2%-81.7%) |
| Male | 217 | 135 | 380 | 74.9% (71.0%-78.9%) |

*CI: confidence interval; NSCLC: non-small cell lung cancer, includes “Other specified carcinoma”.*

*^a^ Anti-cancer treatment includes surgery, chemotherapy and radiotherapy as defined by Table B, from one month prior to cancer registry diagnosis date onwards.*

*^b^ Spread of disease at diagnosis as reported by the New South Wales Cancer Registry.*
