# Supplementary material for: Health services costs for lung cancer care in Australia: Estimates from the 45 and Up Study
Source: PLoS One. 2020 Aug 31;15(8):e0238018. doi: 10.1371/journal.pone.0238018 (PMC7458299; doi:10.1371/journal.pone.0238018)
Supplement: Attachment — Submitted filename: Response to Reviewers.docx [file pone.0238018.s002.docx]

Thank you for the reviews and for the opportunity to submit a revised manuscript. The reviewers’ comments are addressed in detail below. However, we first want to clarify the purpose of the research described in the manuscript and the importance of the results, as this addresses a few of the comments.

The primary aim of the research was to estimate the existing costs of lung cancer care in Australia. These data are currently in demand as key inputs for cost-effectiveness evaluations of lung cancer screening, which is a live policy issue in Australia, and will be valuable for assessing the cost implications of other lung cancer control interventions including tobacco control strategies and new treatment technologies. This has been articulated more clearly and given greater emphasis in the revised manuscript. While we believe these results are of interest to a range of stakeholders, even if they were not, we note that *PLOS ONE* has a policy to “evaluate submitted manuscripts on the basis of methodological rigor and high ethical standards, regardless of perceived novelty”.

**Notes regarding journal requirements**

*When submitting your revision, we need you to address these additional requirements:*

*1. Please ensure that your manuscript meets PLOS ONE's style requirements, including those for file naming.*

Response: The submission has been updated accordingly.

*2. In your ethics statement in the manuscript and in the online submission form, please provide additional information about the patient records used in your retrospective study. Specifically, please ensure that you have discussed whether all data were fully anonymized before you accessed them.*

Response: We have included the following statement in the first paragraph of the Materials and Methods section, and in the online submission form: “All data were fully anonymised before being made available for analysis.”

*3. To comply with PLOS ONE submission guidelines, in your Methods section, please provide additional information regarding your statistical analyses.*

Response: The manuscript now includes the following information on statistical analyses in the Materials and Methods section, as per PLOS ONE’s expectations for statistical reporting.

“Means of excess costs were calculated, along with the proportions of excess costs contributed by each source (inpatient hospitalisations; ED presentations; prescription medicines; other subsidised services in the MBS). Medians, standard deviations and inter-quartile ranges are reported in the Supporting Information (Table A in Supporting information). Analysis was carried out using SAS v9.4 (SAS Inc., NC, US).”

“To assess the independent effects of these factors, we also analysed the costs using gamma regression with a log-link (16), including all factors as covariates, with the effects considered statistically significant when p<0.05. A small number of outlying excess costs were excluded from the regression analysis if they had a ~~(~~standardised Pearson residual of <-4 or >5~~)~~. Due to the potential for negative excess costs, all cost estimates were “shifted” by +$100,000 for this specific analysis so that all were >$0.”

“Where possible, missing values were imputed from information for the same person recorded in other datasets.”

“People with no information for the matching variables were excluded…”

*4. We note that you have indicated that data from this study are available upon request. PLOS only allows data to be available upon request if there are legal or ethical restrictions on sharing data publicly. … In your revised cover letter, please address the following prompts:*

*a) If there are ethical or legal restrictions on sharing a de-identified data set, please explain them in detail (e.g., data contain potentially identifying or sensitive patient information) and who has imposed them (e.g., an ethics committee). Please also provide contact information for a data access committee, ethics committee, or other institutional body to which data requests may be sent.*

*b) If there are no restrictions, please upload the minimal anonymized data set necessary to replicate your study findings as either Supporting Information files or to a stable, public repository and provide us with the relevant URLs, DOIs, or accession numbers.*

Response: The data are available upon request from the relevant data custodians, not from the authors. As noted in the original submission, we are not permitted to publish the data that were used in this study or make the data available to other researchers. We have revised the data availability statement to clarify this as shown below.

“The data cannot be made available by the authors. These are third party data not owned or collected by the authors and on-provision is not permitted by the relevant data custodians (Sax Institute, Services Australia, NSW Ministry of Health), as it would compromise the patients' confidentiality and participants' privacy. The data contain potentially identifying and sensitive patient information. However the data are available from the data custodians for approved research projects - data access enquiries can be made to the Sax Institute (see <https://www.saxinstitute.org.au/our-work/45-upstudy/governance/> for details). Other researchers would be able to access these data using the same process followed by the authors.”

**Responses to comments from Reviewer #1**

*Comment: 1. The study appears to be insufficiently motivated, in that the stated purpose cannot logically be fulfilled by the results of the study. The study justifies its primary aim with the following sentence: “Given the large number of new cases every year, information about changing costs for lung cancer treatment is crucial to health system planning in Australia and in other developed countries.”*

Response: The primary aim of this research was to estimate the costs of lung cancer care in Australia, with comparisons across key patient subgroups. As described above, these data are currently in demand as key inputs for cost-effectiveness evaluations of lung cancer screening, which is a live policy issue in Australia and other high-income settings in Europe and North America. The data will also be valuable for assessing the cost implications of other lung cancer control interventions including tobacco control strategies and new treatment technologies. This has been made clearer and given more emphasis as shown below. We have also included an additional table in the supporting information (Table E) providing summary costs that were requested for a current cost-effectiveness evaluation of lung cancer screening in Australia.

Abstract, Background: “Our aim was to provide detailed population-based estimates of health system costs for lung cancer care, as a benchmark prior to wider availability of targeted therapies/immunotherapy and to inform cost-effectiveness analyses of lung cancer screening and other interventions in Australia.”

Introduction, paragraph 1: “The poor prognosis and healthcare resource requirements mean there is a need for cost-effective interventions aimed at primary prevention, screening, early detection, and improvements in treatment. Costing studies are key to informing cost-effectiveness evaluations, informing policy decisions for cancer control and improving public health.”

Introduction, paragraph 4: “To assess the potential impact of both existing and emerging lung cancer interventions on future healthcare costs, information is needed on the distribution of lung cancer care costs for key patient subgroups, referred to as the “usual care” scenario in cost-effectiveness analyses.”

*Comment: a. First, there is no justification why Australia’s experience can guide health system planning in other developed countries. In fact, very little is given to highlight essential features of the Australian healthcare system, so that an international reader from another developed nation may not necessarily know how applicable lessons learned from this study would be to his or her national setting.*

Response: We have revised the manuscript to make this clearer, as shown below.

Introduction, paragraph 1: “While health services costs are setting-specific, the cost-effectiveness evaluations they inform can be translated across populations with similar healthcare systems by identifying the key drivers of effectiveness and cost-effectiveness. ~~Given the large number of new cases every year, information about changing costs for lung cancer treatment is crucial to health system planning in Australia and in other developed countries (5, 6).~~”

Introduction, paragraph 3: “Changes in costs over time can be quantified using this method in settings like Australia where there is a universal healthcare system with linked records across healthcare sectors or central recording of all encounters with the healthcare system, or where large health funds cover all costs of healthcare for a broad cross-section of the population.”

Introduction, paragraph 4: “Detailed costs for lung cancer care are also timely given the policy setting in Australia and other high-income countries, with the demonstration of lung cancer screening effectiveness in two key trials (20, 21).”

*Comment: b. The primary innovation from the study is stated to be the subgroup analyses in terms of how costs are generated for cancer care in Australia. This sentence (1 above) does not clearly speak to the need for subgroup analyses.*

*Comment: c. Related to point (b) above, there was no explanation/justification as to why the specific subgroups were chosen for analyses. This justification should be tied into the motivation for the study.*

Response: We have revised the manuscript to more clearly address these points, as shown below.

Introduction, paragraph 3: “Individual patient care and the related costs vary based on patient and tumour characteristics. To date, little has been reported on population-based costs for patient subgroups in Australia, in part due to a lack of available data. … We previously used this method to report overall costs for each of the ten most common cancer types in Australia, including lung cancer (4), but did not disaggregate these for important patient subgroups.”

Introduction, paragraph 4: “Stage-specific cost estimates related to lung cancer incidence (e.g. age, smoking status) and factors related to treatment decisions (e.g. histological type, accessibility of services) are needed to update cost-effectiveness evaluations of lung cancer screening in Australia (22-24).”

Introduction, final paragraph: “We estimated lung cancer costs for several patient and tumour subgroups that are likely to be relevant to cost-effectiveness evaluations of lung cancer interventions prior to the wider introduction of new therapies and technologies.”

Statistical analysis, paragraph 2: “Cases were classified by … . These factors were chosen due to their potential association with lung cancer incidence and treatment, as these are key factors relating to a potential lung cancer screening program and other potential interventions (22, 35).”

Discussion, paragraph 1: “To our knowledge this is the first study to provide detailed health system costs for lung cancer care in Australia, stratified according to various patient and tumour characteristics and using population-based estimates from individual-level data. This gives a unique insight into the factors associated with healthcare costs for lung cancer. These results provide key inputs for cost-effectiveness analyses of both existing and emerging lung cancer interventions, including lung cancer screening, tobacco control strategies, and new treatment technologies. We found substantial variation…”

*Comment: d. In fact, lines 75-77 actually provide the information that I was hoping to find earlier on in the introduction. I suggest that the authors move this critical sentence higher up: “It is important to establish methods for estimating overall costs of treatment over time, and to benchmark tumour-specific treatment costs prior to the wider introduction of new targeted therapies and the accompanying molecular testing.” But then see point 2 below.*

Response: As described in the previous responses, we have substantially revised the Introduction to address these comments, clarifying and highlighting the study aims and key uses, along with the reasons for the various analyses.

*Comment: 2. The second issue also relates to motivation. The manuscript states, in lines 75-77 (see 1d above), that we need to benchmark costs prior to wider introduction of new targeted therapies. However, the costs that are estimated and documented are actually costs prior to the new targeted therapies, so they have no direct relevance with the costs of the newer therapies. I find it difficult to understand the rationale for understanding the costs of “old technologies” before introducing “new technologies.” In fact, in line 83-84, the manuscript again notes “to understand future costs, we need to know the costs of key patient groups.” But I am not sure how knowing the costs of current technology for key patient groups will inform future costs, at least not without further explanation.*

Response: We have revised the manuscript to clarify these points. We are estimating the “existing” costs of care during the study period. Knowing these costs, estimated prices for new drugs can be used to estimate the likely costs of care with new technologies (e.g. genetic testing), medicines (e.g. targeted therapies) or detection methods. Combined with estimates of relative effectiveness, this would allow for estimates of cost-effectiveness (weighing up the additional costs versus the additional benefits for the new technology) before wide implementation and collection of new data, and hence could inform decisions regarding implementation and reimbursement.

Within the study period there had been little uptake of targeted therapies as they were applicable only to small subgroups of lung cancer cases. We found that only 4% of cases had a subsidised targeted therapy, with low uptake also noted in a government report regarding medicine utilisation (see below). However, these new technologies and medicines are being made available to a wider range of patients, so future costs will need to look at additional costs incurred for these new technologies and medicines. This will be required to assess the cost-effectiveness of new technologies (e.g. genetic testing), new medicines (e.g. immunotherapy) and lung cancer screening. We have clarified this as shown below.

Introduction, paragraph 4: “To assess the potential impact of both existing and emerging lung cancer interventions on future healthcare costs, information is needed on the current distribution of lung cancer care costs for key patient subgroups, referred to as the “usual care” scenario in cost-effectiveness analyses. For example, since 2013, a number of targeted therapies/immunotherapies (e.g. gefitinib, erlotinib, nivolumab) have been ~~are now~~ subsidised by the Australian government for the treatment of an expanding range of indications relating to lung cancer (17), and molecular testing to determine access to such therapies is increasingly performed. However, uptake was still relatively low during the period described in this study (18). These new methods of cancer care have the potential to improve survival and quality of life, but they are expensive (19), and the broader impact of these new treatment technologies on the health system remains unclear.”

Introduction, final paragraph: “We estimated lung cancer costs for several patient and tumour subgroups that are likely to be relevant to cost-effectiveness evaluations of lung cancer interventions prior to the wider introduction of new therapies and technologies.”

*Comment: 3. The third issue again relates to motivation: If the rationale for the study is to understand the costs of care prior to adopting new technology, why is it important to understand the excess costs due to lung cancer (relative to patients with no lung cancer)? Should we need to know just how much it costs to treat lung cancer; not “how much more” lung cancer costs relative to someone who is similar, but has no lung cancer? In fact, the decision to define “excess” costs also leads to downstream issues, such as those identified in lines 227-229 (the need to shift costs to avoid negative excess costs). If we simply used actual costs (not excess costs), there would be no need to account for negative excess costs.*

Response: The linked administrative data used in this study have good coverage of healthcare utilisation, but with these data it is not possible to directly identify all healthcare that is specific to lung cancer. Some services, such as GP consultations, do not include clinical details that would make them identifiable as healthcare relating to lung cancer, so it is not possible to accurately estimate the “actual costs”. By first including all healthcare utilisation we are able to capture the costs of the healthcare consequences of lung cancer as well as those directly related to treating the disease. The excess costs method then adjusts for other background healthcare costs through matching to cancer-free controls – in our study we matched by age, sex, geographical region and smoking status. In this way, any general variation in costs due to these factors that is not related to cancer will be accounted for. We can then analyse the costs attributable to lung cancer rather than also including the costs that might result from these other factors, as has been established in other cited studies (Blakely et al., Mariotto et al.). Ultimately, this method provides an efficient and reliable estimate of the total burden on health services.

The relatively large sample size means that there will be some random variation that includes negative excess costs (some in the control group might have conditions requiring intensive treatment) and these will be countered by randomly high excess costs. The regression analysis is able to deal with the negative values that result from this natural variation.

*Comment: 4. There may be some confusion as to how “population-level” this study is. The abstract states that the study is “population-level,” but the methods section actually tells a different story – that information was gathered from a trial (that oversamples certain groups); and these patients were selected to participate in a study/survey. Lines 215-27 suggest that the study had to be re-weighted in order to match population-level data. So I would refrain to qualify this study as “population-level.”*

Response: There was no reference to this being a “population-level” study, we have described it as “population-based”. This description is to indicate that it comes from a population-based sample, as opposed to, for example, a hospital-based sample or people who had surgery. As noted in the Discussion section: “We used empirical data from a large population-based sample with detailed individual-level data and included all cases without restriction to a specific hospital catchment or treatment types.” The only mention of “population-level” was in the Discussion where we said that “We weighted the cohort to match the population-level stage distribution”.

The 45 and Up Study participants are from a random sample of the state-wide population, with participants accounting for 10% of the state’s population aged 45 or more (<https://www.saxinstitute.org.au/our-work/45-up-study/>). Our analysis included 45 and Up Study participants who were diagnosed with lung cancer. There was no trial involved. We have acknowledged that the elderly and rural people were oversampled, but the cohort has been shown to be reliable for comparative/relative results (see Mealing et al. reference). As we demonstrate in Table 1, the lung cancer cases were reasonably representative of cases across the state, and there were only minor adjustments required through weighting to the population-level stage distribution.

*Comment: 5. In the study sample section (starting on line 130), there are certain exceptions that are self-evident; but other exclusions are not so clear. For example, why exclude keratinocyte cancers?*

Response: Keratinocyte (non-melanoma skin) cancers were not excluded, this has been clarified in the manuscript. Keratinocyte cancers are not recorded in the cancer registry and are difficult to identify via self-report. They are extremely common in Australia and generally not dangerous, requiring relatively minor treatment to resolve. We have explained the other exclusions in the manuscript and have added the reason for excluding one other subgroup.

Materials and Methods, paragraph 2: “The Centre for Health Record Linkage (28) linked other health records for study participants including … statutory ~~cancer~~ notifications of cancer except ~~excluding~~ keratinocyte (non-melanoma skin) cancers from the NSW Cancer Registry”.

Materials and Methods, paragraph 3: “We excluded participants with: … self-reported history of cancer (except ~~excluding~~ keratinocyte cancers)”

Discussion, paragraph 6: “We excluded 2% of cases who were first diagnosed on their death certificate, as they could not have treatment after diagnosis, along with a small number of cases whose month of diagnosis was not recorded, as their healthcare use could not be accurately assigned to time periods relative to diagnosis.”

*Comment: 6. I am curious about the matching approach that includes matching based on smoking status, particularly matching based on current smoking status. If a case who has smoked for 20 years must be matched with a control who has smoked for 20 years without cancer, this would seem to introduce some odd selection issue.*

Response: The aim of matching by smoking status is to control for the healthcare cost impact of smoking and related comorbidities, so that any cost differences are more likely to be due to lung cancer. The case and their matched controls will generally have healthcare issues relating to their smoking history and the matching aims to remove these background healthcare costs.

*Comment: 7. Finally, I would like to reiterate the importance of establishing the importance of identifying certain subgroups to compare costs. As currently written, the article reads like a collection of facts. I understand that not all manuscripts are hypothesis-driven and therefore will not necessarily progress in a linear fashion. However, a descriptive study that presents a lot of results by slicing the data in different ways actually ends up being difficult to read. Perhaps motivating the reasons for looking at costs for different subgroups may help lessen this sense?*

*I.e., assessing costs by cancer stage seems quite appropriate. But what about all the statistics presented under “average costs”? Or facts and figures under “monthly costs”? Are not monthly costs highly correlated with the stage of cancer?*

Response: As described above, we have substantially revised the manuscript (particularly the Introduction) to clarify the reasons for estimating costs by these factors, highlighting their importance for assessing the cost-effectiveness of lung cancer screening, a live policy issue in Australia, along with other lung cancer control interventions. We also want to give a broad picture of the costs of lung cancer care per patient, and how costs differ using various time perspectives relative to diagnosis, providing data that are potentially useful to other researchers. These provide a wider picture before being disaggregated for subgroups, where we can provide a more in-depth picture of costs for lung cancer care. We recognise that this can be a lot of information for a paper so we have tried to put most of the highly detailed tables in the supporting information file.

*Comment: Also, the exploratory analyses of new cases from 2014-2015 seem ad hoc – They seem to be an afterthought because they are not sufficiently developed relative to the other sections.*

Response: It was not possible for this exploratory analysis to be as well developed based on the available data, and this is addressed in the study limitations. However, the data do provide important real-world information regarding the potential changes in costs of lung cancer care based on new and expensive technologies and treatments. We believe it is important to include this information in this context and have noted that in the Discussion. As described previously, we report that within the main study period there had been little uptake of targeted therapies as they only applied to small subgroups of lung cancer cases.

Discussion, paragraph 4: “However, identifying cases this way does not capture all cases and histology/staging information was not available to allow more informed analyses of these cases. This will be an area for further investigation as more recent NSWCR data become available. However, these data are still informative, marking the beginning of an upward trend in the use of new technology and medicines in real-world lung cancer care.”

*Comment: 8. Without ending on a morbid note, the final paragraph indirectly suggests that a cost-saving technique in cancer care is to diagnose later. I would suggest rewording the last paragraph, so that the paragraph does not begin with noting that “lower costs are associated with shorter survival time … and this is important information for health services planning.”*

Response: We have reworded the last paragraph as shown below.

Conclusions: “The costs of lung cancer care are substantial and are lower, on average, for people with features associated with poorer survival. … A key aim of future health services planning for lung cancer is to improve survival, and it is important to know about the potential cost implications. These cost estimates provide important information for health services planning and delivery and the evaluation and implementation of new interventions for cancer control. Costs are likely to grow with the wider availability of new therapies and technologies, and increasing survival times.”

*Minor comment 1. What does it mean that ED costs were assigned based on triage category and discharge status? Why not just simply tally up all the costs during a specific ED visit?*

Response: Unfortunately, the available data do not allow this approach to obtain ED costs. As with the hospitalisations, we assigned the individual episode costs according to the nationally-calculated average cost for that type of healthcare episode. In the case of ED visits the cost classification is done by triage category and discharge status. We have clarified the wording accordingly.

Materials and Methods, Data for costs, paragraph 3: “~~Costs f~~For ED presentations, there was insufficient treatment information available to assign detailed costs so we used average costs from the 2010 National Hospital Cost Data Collection, ~~were~~ assigned according to triage category and discharge status (30).”

*Minor comment 2. Line 303 seems to be contradicted by the table: Isn’t the cost for current smoker lower? The text says costs for current smokers are higher.*

Response: Thank you for pointing this out, the text has been corrected accordingly.

“Higher costs were associated with younger age at diagnosis (versus lower costs for cases aged ≥80), while lower costs were associated with current smoking at study recruitment, …”

**Responses to comments from Reviewer #2**

*Comment: The manuscript relies on descriptive stats and simple mean comparisons of matched samples to analyze the ‘costs’ of lung cancer treatment in Australia. The authors find that as expected the cost of lung cancer treatment is lower for older patients, varies across different types of tumor histology, and is strongly inflationary. That is all rather trivial.*

*The authors also find an inverse u-shaped relation between socioeconomic status and treatment costs. Patients from most and least deprived socioeconomic strata are on average less costly. This is interesting, but the authors do explain these findings. One has to say that the manuscript remains unanalytical and simply describes differences without trying to explain them.*

Response: As described in the responses above, we have made several revisions to the manuscript to clarify and emphasise the study aims, with substantial changes to the Introduction in particular. This study provides descriptive data on costs for use in cost-effectiveness analyses for lung cancer control interventions, helping to inform future healthcare planning. It is not our aim to address novel hypotheses. These data are currently in demand and we have included an additional table in the supporting information (Table E) providing summary costs that were requested for a current cost-effectiveness evaluation of lung cancer screening in Australia.

In the discussion section we do explain our findings based on age, smoking status, cancer histology/staging and phase of care. We did not go into detail about the lower costs for people from less disadvantaged areas as this only just reached statistical significance at the p<0.05 level in one of the four regressions and was not a repeated trend as was found for the other factors.

*Comment: From a methodological perspective I do not believe that patient characteristics are uncorrelated. Therefore, most of the reported mean differences will be biased if the treatment and control group are not perfectly matched in all relevant dimensions. What is clearly missing, to give an example, are the existence of diseases other than lung cancer.*

Response: We have matched cases with multiple controls by age, sex, geographical area and smoking status. We could identify those comorbid conditions self-reported at baseline, but it becomes difficult to find sufficient numbers of matched controls when these additional characteristics are included. It would also raise the potential for over-matching, along with the potential incorrect use of factors on the pathway to a lung cancer diagnosis. The matching process we have used should broadly account for the key differences between the cases and controls, other than their lung cancer status. In particular, matching by smoking status should account for similarities in comorbidities. The matching of multiple controls per case also adds to the robustness of the results.

*Comment: In sum, I find the manuscript borderline publishable. I also cannot see many implications. Yes, the costs of lung cancer treatments differ systematically, but so what?*

Response: As described in earlier responses, we have revised the manuscript to clarify and emphasise the aims and implications of the manuscript. The primary aim is to establish the existing costs of lung cancer care in Australia. These data are currently in demand to assess the cost-effectiveness of lung cancer screening, which is a live policy issue in Australia and other high-income countries. The results are also valuable for assessing the cost implications of other lung cancer control interventions including tobacco control strategies and new treatment technologies. We have included an additional table in the supporting information (Table E) providing summary costs that were requested for a current cost-effectiveness evaluation of lung cancer screening in Australia. While we believe these results are of interest to a range of stakeholders, even if they were not, we note that *PLOS ONE* has a policy to “evaluate submitted manuscripts on the basis of methodological rigor and high ethical standards, regardless of perceived novelty”.

*Comment: I also find it implausible that the data cannot be made publicly available. This should be a reason to reject the paper. If data is not shared and no replication policy in place, it is not science.*

Response: As noted in our submission, we cannot make the data publicly available due to restrictions placed upon us by the data custodians – this was a condition of the release of data to researchers. However, other researchers can access the data from the data custodians if they go through the relevant application process. This has been made clearer in our revised submission.

“The data cannot be made available by the authors. These are third party data not owned or collected by the authors and on-provision is not permitted by the relevant data custodians (Sax Institute, Services Australia, NSW Ministry of Health), as it would compromise the patients' confidentiality and participants' privacy. The data contain potentially identifying and sensitive patient information. However the data are available from the data custodians for approved research projects - data access enquiries can be made to the Sax Institute (see <https://www.saxinstitute.org.au/our-work/45-upstudy/governance/> for details). Other researchers would be able to access these data using the same process followed by the authors.”

**Responses to comments from Reviewer #3**

*Comment: … However, after knowing those different costs, one could ask the question of “so what?”. They may be the need to relate these costs to the standard of living of those patients. From the Australian standard is it something that an average individual can afford, or some measure need to be put in place to support patients? This is important to better inform policymaking.*

Response: As described in earlier responses, we have revised the manuscript to clarify that our primary aim is to establish the existing costs of lung cancer care in Australia. These data are needed to assess the cost-effectiveness of lung cancer screening, which is a live policy issue in Australia and other high-income countries, as well as for assessing the cost implications of other lung cancer control interventions including tobacco control strategies and new treatment technologies.

We have also revised the manuscript to indicate that the methods may be applicable to regions outside of Australia.

Introduction, paragraph 1: “While health services costs are setting-specific, the cost-effectiveness evaluations they inform can be translated across populations with similar healthcare systems by identifying the key drivers of effectiveness and cost-effectiveness.”

Introduction, paragraph 3: “Changes in costs over time can be quantified using this method in settings like Australia where there is a universal healthcare system with linked records across healthcare sectors or central recording of all encounters with the healthcare system, or where large health funds cover all costs of healthcare for a broad cross-section of the population.”

Introduction, paragraph 4: “Detailed costs for lung cancer care are also timely given the policy setting in Australia and other high-income countries, with the demonstration of lung cancer screening effectiveness in two key trials (19, 20).”

In terms of what an average individual can afford, we are reporting costs to the health system and so the affordability to the individual is not directly relevant here. That would be for consideration in a study of patients’ out-of-pocket costs and as described in the manuscript, information on the out-of-pocket costs was not available.
